# An extensive analysis of disease-gene associations using network integration and fast kernel-based gene prioritization methods

**DOI:** 10.1016/j.artmed.2014.03.003

**Published:** 2014-06

**Authors:** Giorgio Valentini, Alberto Paccanaro, Horacio Caniza, Alfonso E. Romero, Matteo Re

**Affiliations:** a AnacletoLab – Dipartimento di Informatica, Università degli Studi di Milano, via Comelico 39/41, 20135 Milano, Italy; b Department of Computer Science and Centre for Systems and Synthetic Biology, Royal Holloway, University of London, Egham TW20 0EX, UK

**Keywords:** Gene disease prioritization, Network integration, Heterogeneous data fusion, MeSH descriptors, Node label ranking

## Abstract

**Objective:**

In the context of “network medicine”, gene prioritization methods represent one of the main tools to discover candidate disease genes by exploiting the large amount of data covering different types of functional relationships between genes. Several works proposed to integrate multiple sources of data to improve disease gene prioritization, but to our knowledge no systematic studies focused on the quantitative evaluation of the impact of network integration on gene prioritization. In this paper, we aim at providing an extensive analysis of gene-disease associations not limited to genetic disorders, and a systematic comparison of different network integration methods for gene prioritization.

**Materials and methods:**

We collected nine different functional networks representing different functional relationships between genes, and we combined them through both unweighted and weighted network integration methods. We then prioritized genes with respect to each of the considered 708 medical subject headings (MeSH) diseases by applying classical guilt-by-association, random walk and random walk with restart algorithms, and the recently proposed kernelized score functions.

**Results:**

The results obtained with classical random walk algorithms and the best single network achieved an average area under the curve (AUC) across the 708 MeSH diseases of about 0.82, while kernelized score functions and network integration boosted the average AUC to about 0.89. Weighted integration, by exploiting the different “informativeness” embedded in different functional networks, outperforms unweighted integration at 0.01 significance level, according to the Wilcoxon signed rank sum test. For each MeSH disease we provide the top-ranked unannotated candidate genes, available for further bio-medical investigation.

**Conclusions:**

Network integration is necessary to boost the performances of gene prioritization methods. Moreover the methods based on kernelized score functions can further enhance disease gene ranking results, by adopting both local and global learning strategies, able to exploit the overall topology of the network.

## 1 Introduction

The raising awareness that a disease is rarely a consequence of an abnormality on a single gene, but it is usually the result of complex interactions and perturbations involving large sets of genes and their relationships with several cellular components, lead to development of the “Network medicine”, a network based approach to human disease [1]. In this context, gene prioritization methods have progressed quickly with the aim of discovering candidate “disease” genes by exploiting the large amount of available “omics” data covering different types of relationships between genes [2].

According to [3], automatic gene prioritization methods typically produce their outputs either by filtering the candidate genes into smaller subsets or by ranking the candidate genes.

*Filtering methods* are based on the definition of a set of criteria motivated by the available knowledge of the molecular basis of the disease under investigation. Their main objective is to reduce the set of potential disease genes by exploiting a comparison of all the candidate genes with a sort of gene template, which encodes the selection criteria in a set of rules [4,5]. Despite having been proved effective [6,7], the hard filtering policy underlying their functioning is a double-edged sword. Indeed, when a relevant gene fails to meet just one of the criteria encoded in the filter, it becomes a false negative, and this prevents the ability to detect genes that are actually involved in the disease, but with mechanisms not been previously reported in literature.

The second class of gene prioritization methods (*ranking based*) avoids the limitations of filtering methods simply by ranking candidates from most to least promising ones. As in the case of filtering methods, ranking based methods can integrate multiple sources of evidence in the gene prioritization process. These methods can be further classified into three main categories [3]: text mining [8,9], similarity profiling and network analysis-based [10–13].

Although powerful in their ability to make a very effective usage of the available knowledge, text mining approaches show a strong bias toward the identification of straightforward candidates for which abundant knowledge is already available [14]. On the contrary, similarity profiling [15] and network analysis based gene prioritization systems are not affected by this limitation. Indeed they can exploit both knowledge bases (increasing the specificity of predictions) and raw data (for novel predictions).

In particular, network based methods are gaining increasing popularity in disease gene prioritization (see [16,17] for recent specific reviews). According to this approach, nodes represent genes and edges encode some notion of functional similarity between genes, e.g. direct molecular interactions, transcriptional co-expression/regulation, sequence or structure similarity or paralogy [18]; the prioritization list is then constructed by exploiting the topology and the edge weights of the network and a set of “core” genes known to be associated to the disease under study. In this category some methods used a random walk or a heat kernel [19], while others applied Web and social networks methods on a protein–protein interaction (PPI) network [20], and other approaches exploited PPI and pathway information to prioritize candidate genes [21,15].

Most gene prioritization methods exploited different sources of information and gene networks [22,23], ranging from phenotypic similarities between diseases and functional similarity between genes [24], to GO ontology and InterPro domain annotations [25] and protein–protein interactions, gene expression and common membership to KEGG pathways [26], and also to several other sets of data sources [15,27,28] (see [22] for a more detailed presentation of the different combinations of sources of evidence exploited by recent disease genes prioritization methods).

Despite the large availability of works describing specific combinations of datasets to develop tools suitable for disease genes prioritization, “our understanding of how to perform useful predictions using multiple data sources or across biological networks is still rudimentary” [3], and in particular, to our knowledge, no systematic studies focused on the comparison of different network integration methods.

To contribute to fill this gap, in this paper we propose, compare and analyze different network integration strategies to combine multiple gene networks constructed with different sources of single or heterogeneous data. In particular we apply simple unweighted integration methods, that combine gene networks solely on the basis of the structural characteristics of the nets, and we propose weighted integration methods that combine networks according to the “predictiveness strength” of each type of network, estimated through the assessment of the accuracy of the learning algorithm trained on each of the combined networks. We constructed and integrated nine different gene networks, including also semantic similarity-based gene networks, since it has been recently shown that they improve gene-disease prioritization [29,30].

Another contribution of this work consists in the application of the *kernelized score functions* to the gene-disease prioritization problem. This novel semi-supervised network method for node label ranking adopts both local and global learning strategies to learn from both the neighborhood of each node and at the same time from the overall topology of the network [31,32].

Another open issue is represented by the choice of the “seed genes” to characterize the diseases involved in the gene prioritization analysis [22]. Previous methods focused on specific diseases [33,34] or on genetic diseases [23,35] according e.g. to the online Mendelian inheritance in man (OMIM) database [36]. In order to extend the analysis to a larger set of diseases, not limited to genetic disorders, in this work we used “seed genes” borrowed from the MeSH taxonomy of diseases [37], by exploiting gene-MeSH disease associations provided by the comparative toxicogenomics database (CTD) [38].

Summarizing, our main contributions can be schematized as follows:

• We propose one of the widest gene-disease prioritization studies, involving gene-MeSH disease associations covering more than 700 diseases, not limited to genetic disorders.
• We propose novel weighted integration methods able to combine multiple networks according to the “predictiveness strength” of each source of data.
• A comparative analysis of different network-integration methods, and a quantitative evaluation of their impact on gene-disease prioritization.
• An extensive application of the *kernelized score functions*, a recently proposed semi-supervised network-based method that embeds local and global learning strategies, to the gene disease prioritization problem.

This paper is structured as follows. In Section 2.1 we introduce MeSH and the pipeline we applied to annotate the “seed genes” used in our experiments. Section 2.2 describes the functional networks considered in our experiments. Then in Section 2.4 the unweighted and weighted integration methods and in Section 2.5 the gene prioritization methods used in our experiments are introduced. The overall experimental setting is described in Section 3.1, and the results relative to the application of the gene prioritization methods to the single functional networks are discussed in Section 3.3. These results are then quantitatively compared with those obtained through unweighted (Section 3.4) and weighted (Section 3.5) network integration methods, while in Section 3.6 the top-ranked unannotated genes and the AUC and *p*-value associated to each of the 708 MeSH diseases analyzed in this work are presented. The conclusions outline the main findings of this work and suggest novel research lines in the context of the gene prioritization and network integration problems.

## 2 Materials and methods

### 2.1 MeSH: medical subject headings

MeSH is a controlled vocabulary produced by the National Library of Medicine for indexing, cataloging, and searching biomedical and health-related information and documents (http://www.nlm.nih.gov/mesh, accessed 30 November 2013). The descriptors or subject headings of MeSH are arranged in a hierarchy. MeSH covers a broad range of topics and its current version consists of 16 top level categories. The MeSH thesaurus is used for indexing articles from the world's leading biomedical journals for the MEDLINE/PubMED database. One of the MeSH top level terms (Diseases) is used to label the gene sets used in our experiments and to evaluate the impact of network integration on the inference of relationships between genes and diseases.

The associations between the genes and the MeSH disease terms have been downloaded from the CTD [38], a public resource that provides information about the interaction of environmental chemicals with gene products and their effects on human diseases. These relationships are annotated from the scientific literature by professional biocurators who manually curate a triad of core interactions including chemical-gene, chemical-disease and gene-disease relationships. The CTD integrates these core data to generate inferred chemical-gene-disease networks.

**Fig. 1:**
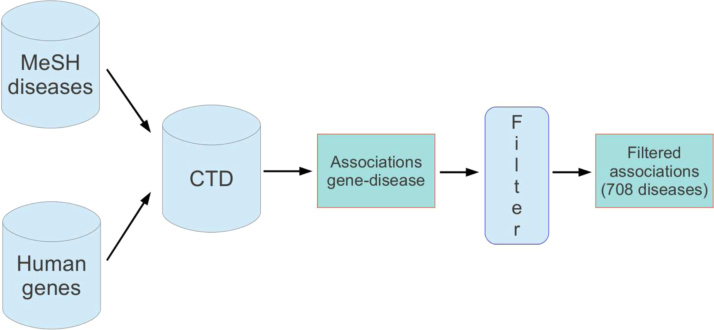
Pipeline of the gene – MeSH disease annotation process.

To provide a “gold standard” of “seed genes” to infer novel gene-disease associations, we first downloaded the associations between the human genes considered in our experiments (Section 2.2) and all the available MeSH disease terms available in CTD. We then filtered out all the diseases associated with less than five and more than 200 genes in order to both ensure a minimum amount of a priori information for our prediction tasks and to avoid classes whose associated gene sets are too heterogeneous. This led to the definition of a set composed by 708 MeSH diseases (Fig. 1).

The full set of the “gold standard” seed genes – MeSH disease associations is available from http://homes.di.unimi.it/valentini/DATA/DiseaseGeneNetworks (accessed 30.11.13).

It is worth noting that MeSH controlled vocabulary of diseases has been just proposed in the context of text-mining-based gene prioritization [39], but those results cannot be safely generalized to network-based methods, since text-mining approaches show a bias toward genes for which a large “a priori” knowledge is actually available in literature [14].

### 2.2 Functional networks

We collected different sources of data to represent different functional relationships between genes. More precisely, we constructed gene networks using physical and genetic interactions, transcriptional co-expression/regulation and localization, protein domain and gene chemical interactions, co-occurrence of disease-gene pairs in scientific texts, homologues implicated in generating similar phenotypes in other organisms, common molecular pathways between gene products, and common GO annotations.

Table 1 summarizes the main characteristics of the nine gene functional networks used in our experiments. Each gene network includes a set *S* of 8449 genes (or a subset of them) selected according to the procedures described in [40]. We considered a set of genes for which sufficient functional data are available, and for which a relatively comparable coverage across gene networks can be assured. In this way, on the one hand a certain amount of functional information is ensured for each gene, and on the other hand the available information for each considered gene results comparable.

In the rest of this section we provide a brief description of each gene network. The full data sets are downloadable from: http://homes.di.unimi.it/valentini/DATA/DiseaseGeneNetworks (accessed 30.11.13).

#### 2.2.1 Functional interaction network – finet

In [41] Wu and colleagues constructed a functional protein interaction network based on functional interactions predicted by a Naive Bayes classifier trained on pairwise relationships extracted from curated pathways and non-curated sources of information, including protein–protein interactions, gene co-expression, protein domain interaction, Gene Ontology (GO) annotations and text-mined protein interactions. From the original network we extracted the subnetwork including the subset *S* of genes used in our experiments.

#### 2.2.2 Human net – hnnet

Similar in spirit to the approach in [41], the functional network construction method presented in [27] by Lee and colleagues integrates diverse lines of evidence in order to produce a functional human gene network. It has been used in several tests to predict causal genes for human diseases and to increase the power of genome-wide association studies. Also in this case we extracted from Human Net the subnetwork including the subset *S* of genes.

#### 2.2.3 Cancer module network – cmnet

By exploiting gene expression profiling, Segal and colleagues constructed a functional module map for cancer to investigate commonalities and variations between different types of tumor [42]. In their work the authors analyzed a collection of expression profiles with the aim to identify sets of genes that act in concert to carry out specific functions in different cancer types, and then produced a module map constituted by a collection of the gene sets associated to specific cancer gene modules.

**Fig. 2:**
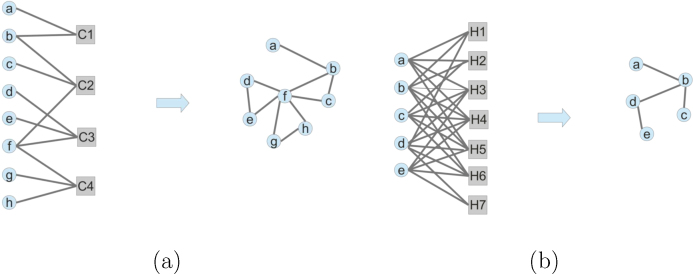
Simplified representation of bipartite network projections into homogeneous gene networks. (a) Binary projection to construct the *cmnet* network; (b) sum projection to construct *gcnet*. Circles represent genes, squares represent cancer modules (a) and chemicals (b).

We used the relationships between the human genes and the Segal's cancer modules [42] to construct a bipartite network. This network has been projected onto the gene space thus originating the *cmnet* network. The type of projection used in the construction of *cmnet* is a *binary* bipartite network projection, meaning that the weight of the edge linking two genes in the projected network is 1 if the two genes share *at least one* neighbour in the original bipartite network and 0 otherwise (Fig. 2a).

#### 2.2.4 Gene chemical network – gcnet

The CTD stores information mined from literature about the interactions between genes, chemicals and diseases in many species. Since one of the objectives of this work is the evaluation of the capabilities of heterogeneous networks integration in the prediction of genes–diseases relationships, we used the genes–chemicals relationships available in the CTD to construct a gene interactions network (*gcnet*). To this end we downloaded from CTD the chemicals–genes interactions file (http://ctdbase.org/reports/CTD_chem_gene_ixns.csv.gz, accessed 30.11.13) and we constructed a bipartite network. We then performed a SUM projection onto the gene space, by which the weight of an edge linking two genes equals the number of the common neighbors of the genes in the bipartite network. The resulting network has finally been binarized using a cutoff of five or more common chemicals interactors to set a binary interaction between a pair of genes (Fig. 2b).

#### 2.2.5 BioGRID database network – dbnet

This is a protein–protein interaction network constructed using direct physical and genetic interactions obtained from BioGRID [43] (v. 3.2.96 – January 2013).

#### 2.2.6 BioGRID projected network – bgnet

Instead of setting-up a binary interaction network based on the direct interaction between the *S* genes, we constructed a bipartite network based on the content of the BioGRID, but using as top nodes the *S* genes and as bottom nodes all the human genes *B* available in BioGRID. More precisely, if in BioGRID does exist an interaction between a node *a* ∈ *S* and *x* ∈ *B*, we added the (*a*, *x*) edge in the bipartite network. Then, according to a binary projection to the *S* space, an edge (*a*, *b*), *a* ∈ *S*, *b* ∈ *S* is added to the projected network if *a* and *b* share at least one common node *x* ∈ *B* in their neighborhoods of the bipartite network. In this way we can capture indirect interactions between pairs of genes.

#### 2.2.7 Semantic similarity-based networks: bpnet, mfnet and ccnet

The last three networks considered in this work have been constructed by computing the Resnik semantic similarities [44] between the terms of each division of the Gene Ontology: biological process, molecular function and cellular component. We obtained a pairwise gene similarity measure by choosing the maximum Resnik semantic similarity between all the terms for which the two genes are annotated. The resulting networks were named *bpnet*, *mfnet* and *ccnet* respectively. The semantic similarity measures have been computed using a MATLAB application implementing methods described in [45].

### 2.3 Basic notation

Gene networks for disease prioritization can be represented through an undirected weighted graph *G* = (*V*, *E*), where *V* is the set of vertices corresponding to genes and *E* the set of edges corresponding to some notion of functional relationship between pairs of genes/vertices. Vertices of the graph and genes can be denoted with natural numbers 1, 2, …, *n*, since each vertex of *G* is univocally associated to a gene. The corresponding adjacency matrix ***W*** with weights $w_{\mathrm{ij}}$ represents the “strength” of the relationship between vertices *i*, *j* ∈ *V*; *V*_*M*_ ⊂ *V* denotes a subset of “positive” vertices belonging to a specific MeSH subject heading *M* (e.g. a MeSH descriptor of a disease – Section 2.1).

We considered the integration of *n* gene networks, *G*^*d*^ = (*V*^*d*^, *E*^*d*^), 1 ≤ *d* ≤ *n*, and we denote by $\bar{G}$ the integrated network $\bar{G}=(\bar{V},\bar{E})$, with $\bar{V}={⋃}_{d}V^{d}$ and $\bar{E}\subseteq {⋃}_{d}E^{d}$. The weights of the edges (*i*, *j*) ∈ *E*^*d*^ are represented with $w_{\mathrm{ij}}^{d}$. Finally a set of features ***x***_*i*_ ∈ *X* can be associated to a gene *i*. For instance, ***x***_*i*_ could represent the genetic or protein interactions, the expression profile or whatever available data for a given gene/vertex *i*.

### 2.4 Network integration methods

We designed and applied different network integration methods to combine different sources of evidence of functional relationships between genes. Our aim consists in providing an analysis of the impact of network integration to gene prioritization, in order to understand whether the combination of multiple networks, constructed from different sources of information, can significantly enhance the performance of gene prioritization methods, and to provide a quantitative assessment of this hypothesized improvement. To this end we programmatically considered relatively simple methods, ranging from unweighted to weighted network integration algorithms, excluding more complex algorithms proposed in the literature, to allows us to perform an extensive analysis involving a large set of diseases, a large set of human genes and a significant subset of the integration methods applied to gene prioritization problems.

Unweighted methods are characterized by networks combinations depending only on the structure of the network itself, while weighted ones depend on an estimate of the learning capabilities of network algorithms or on the assessment of the “informativeness” of the available data. The methods proposed in Section 2.4.2 (unweighted integration) and in Section 2.4.3 (weighted integration) share several general characteristics with previously proposed methods applied in gene prioritization problems or in other computational biology problems such as gene function prediction [46–49].

For instance, unweighted approaches such as the simple union of networks has been applied to the prioritization of genes in Alzheimer's disease using a guilt-by-association inference rule [47], or to the integration of PPI data of model organisms mapped to human through homology [19], or in the context of the functional interpretation of genomic variants to the integration of gene interaction networks [50], or to find functional modules in networks integrated from multiple public databases [51]. Other unweighted approaches for gene prioritization average the scaled Gram matrices obtained from different sources of functional information using suitable kernels [46].

Weighted approaches differ for the way the weights associated to each network are estimated. For instance, weights can be obtained through an iterative algorithm shown to be equivalent to an expectation-maximization (EM) optimization algorithm [52], or weights are learnt by solving a quadratically constrained linear program in a novelty detection setting of the gene prioritization problem [46], or in the context of the gene function prediction problem weights can be interpreted from a probabilistic standpoint [49] or estimated using the PPV (positive prediction value) associated to the edges of the graph [48].

In the following sections, we describe the network pre-processing and the unweighted and weighted network integration methods that we tested in our experiments.

#### 2.4.1 Network pre-processing

Before the combination phase each network underwent a pre-processing step to allow networks for having different number of nodes, to filter some edges in too dense graphs, and to make the weights comparable across different networks. In particular, to deal with genes missing in some networks, we filled the corresponding rows/columns of the symmetric adjacency matrix ***W*** with zeros. To reduce the complexity of the network and the noise introduced by too small edge weights, as a pre-processing step we eliminated edges below a given threshold. In this way we removed very weak similarities between genes, but at the same time we chose relatively low thresholds to avoid the generation of “singletons” with no connections with other nodes. In brief, we tuned the threshold for each network to guarantee that each vertex has at least one connection: for each node/gene we computed the maximum of the weights associated to its edges, and between the selected maxima we chose the minimum as a general threshold for the network. Finally, to make the weights comparable across different networks, avoiding the undesirable effect that a certain network could overcome the others because of the high values of its weights, we applied both Laplacian regularization [53] and a simple linear regularization to obtain weights ${\hat{w}}_{\mathrm{ij}}\in [0,1]$:

(1) $${\hat{w}}_{\mathrm{ij}}=\frac{w_{\mathrm{ij}}-\min_{x,y}w_{\mathrm{xy}}}{\max_{x,y}w_{\mathrm{xy}}-\min_{x,y}w_{\mathrm{xy}}}$$

where indices *x*, *y* ∈ *V* refer to the vertices/genes of the underlying graph.

In our experiments we adopted the regularization shown in (1), since the results were comparable with Laplacian regularization (data not shown).

#### 2.4.2 Unweighted network integration

In the unweighted network integration the combination of different networks depends only on the structure and the characteristics of each network, and no learning is involved in the computation of the integrated network.

##### 2.4.2.1 Unweighted average (UA)

One of the widely applied approach is represented by the *UA* method [46,32]. The weight of each edge of the combined networks is computed simply averaging across the available *n* networks:

(2) $${\bar{w}}_{\mathrm{ij}}=\frac{1}{n}\sum_{d=1}^{n}w_{\mathrm{ij}}^{d}$$

Note that in this integration approach also weights $w_{\mathrm{ij}}=0$ contributes to the average, independently of the fact that the measure of functional relationship between genes *i* and *j* underlying the evidence source is available or not.

##### 2.4.2.2 Per-edge unweighted average (PUA)

We propose a novel method, similar to *UA*, but that assures a high coverage of the genes included in the integrated functional network, without penalizing genes for which a specific source of data is unavailable. With respect to the *UA* method, *PUA* takes into account the fact that a given functional relationship between a pair of genes could be missing, averaging that edge only by the number of networks containing both genes.

More precisely, given a set of *n* gene networks the weight ${\bar{w}}_{\mathrm{ij}}$ of the edge $(i,j)\in \bar{E}$ is computed as follows:

(3) $${\bar{w}}_{\mathrm{ij}}=\frac{1}{\left|D(i,j)\right|}\sum_{d\in D(i,j)}w_{\mathrm{ij}}^{d}$$

where *D*(*i*, *j*) = {*d*|*i* ∈ *V*^*d*^ ∧ *j* ∈ *V*^*d*^}.

##### 2.4.2.3 Network maximum integration (MAX)

The *MAX* integration selects the largest weight among all the available sources of data:

(4) $${\bar{w}}_{\mathrm{ij}}=\max_{d}w_{\mathrm{ij}}^{d}$$

This approach performs the union of all the available sources of evidence [47,51,50], and when multiple edges (*i*, *j*) for a given pair on genes *i* and *j* are available, selects the one with the largest weight.

##### 2.4.2.4 Network minimum integration (MIN)

Analogously, the *MIN* integration selects the minimum weight:

(5) $${\bar{w}}_{\mathrm{ij}}=\min_{d}w_{\mathrm{ij}}^{d}$$

In practice it realizes the intersection between multiple networks. It can be implemented in two different flavours: the “drastic” algorithm (5) for which it is sufficient a single $w_{\mathrm{ij}}^{d}=0$ in order to set ${\bar{w}}_{\mathrm{ij}}=0$, and a “soft” version for which the edges whose weights are set to 0 are discarded, and ${\bar{w}}_{\mathrm{ij}}=0$ if and only if the weights for the edge (*i*, *j*) in all the available networks are set to 0:

(6) $${\bar{w}}_{\mathrm{ij}}=\left\{\begin{array}{cc} 0 & \mathrm{if}\;\forall d\;w_{\mathrm{ij}}^{d}=0\\ \min_{d}\{w_{\mathrm{ij}}^{d}|w_{\mathrm{ij}}^{d}\neq 0\} & \mathrm{otherwise} \end{array}\right)$$

It is worth noting that that this approach could be highly affected by noisy data. It could be reliable when a large evidence is shared among different sources of data.

#### 2.4.3 Weighted network integration

The unweighted methods do not require to learn any parameters from the data, while the weighted integration learns the “weight” *γ* associated to each network. The basic idea behind these approaches consists in associating a *γ* parameter to the “predictiveness strength” of each type of network. This can be realized by using a learning algorithm to associate the “predictiveness strength” of a network with the assessment of the accuracy of the learning algorithm trained on the network itself.

Different weighted approaches have been proposed in the literature [46,52,48,54]. In our experiments, considering that in gene prioritization the main objective consists in effectively ranking the genes with respect to a given disease, we computed the weights according to the AUC obtained for a given MeSH descriptor. More precisely, having *n* networks and *c* MeSH descriptors, we can compute the weight *γ*^*d*^(*k*) for the *d*th network and the *k*th MeSH disease in the following way:

(7) $$\gamma^{d}(k)=\frac{M^{d}(k)}{\sum_{j=1}^{n}M^{j}(k)}$$

where *M*^*d*^(*k*) represents the metric applied to measure the accuracy of the prediction (e.g. the AUC or the precision at a fixed recall) with respect to *k*th MeSH descriptor and the *d*th network. The denominator in (7) simply assures that $\sum_{d=1}^{n}\gamma^{d}(k)=1$. The *γ*^*d*^(*k*) can be computed for each MeSH descriptor *k* by estimating the corresponding AUC by leave-one-out on the training data, that is to say, an “internal” cross validation is performed to optimize the weights, by subdividing each fold of an “external” cross validation applied to evaluate the method in the whole dataset.

##### 2.4.3.1 Weighted average per class (WAP)

By using the *γ*^*d*^(*k*) computed according to (7), the *WAP* method integrates the networks by putting a weight proportional to the performance of a given learning algorithm on each network used in the integration:

(8) $${\bar{w}}_{\mathrm{ij}}(k)=\sum_{d=1}^{n}\gamma^{d}(k)\;w_{\mathrm{ij}}^{d}$$

It is worth noting that in this way we construct a different weighted integrated network for each MeSH descriptor.

In order to emphasize the weight of the most informative networks and, at the same time, to reduce the weights of the least informative ones, a monotonic logarithmic transformation of the weights can be applied, instead of using the one proposed in (7):

(9) $$\gamma^{d}(k)=\frac{\log(1-M^{d}(k))}{\sum_{j=1}^{n}\log(1-M^{j}(k))}$$

We assume that the metric *M* has values in [0, 1] (consider, e.g. the AUC). Note that in a practical implementation, to avoid *γ*^*d*^(*k*)→ ∞, we need to set an upper bound *b* < 1 for *M*. For instance, in our experiments we used the AUC and we set *b* = 0.99.

##### 2.4.3.2 Weighted average (WA)

The *WAP* method adapts the weights *γ*^*d*^(*k*) according to the performance of a learning algorithm on each specific class *k* under study. On one hand, this could lead to a set of networks well fitted to the characteristics of each class *k*, but on the other hand this approach is likely to overfit the data. To this end we introduce a sort of “regularized” version to reduce possible overfitting problems in the learning process. More precisely we compute a regularized weight *γ*^*d*^, by averaging across classes, in the spirit of the approach proposed in [55] in the context of gene function prediction problems. In this way we obtain a unique weight *γ*^*d*^ for each network:

(10) $$\gamma^{d}=\frac{1}{c}\sum_{k=1}^{c}\gamma^{d}(k)$$

The *WA* method, using the weights estimated in (10), builds a unique integrated network, independently of the MeSH disease considered:

(11) $${\bar{w}}_{\mathrm{ij}}=\sum_{d=1}^{n}w_{\mathrm{ij}}^{d}\sum_{k=1}^{c}\frac{\gamma^{d}(k)}{c}=\sum_{d=1}^{n}\gamma^{d}w_{\mathrm{ij}}^{d}$$

Note that in this section we considered the integration of graphs represented through their corresponding adjacency matrices ***W***, but it is easy to see that the same method can be applied to kernel matrices ***K*** derived from ***W***, by simply substituting in each equation the $w_{\mathrm{ij}}$ elements of the adjacency matrix with the *k*_*ij*_ elements of the corresponding kernel matrix (see Section 2.5.1).

### 2.5 Gene prioritization methods

In this section we introduce the gene prioritization methods applied in our experiments. We focused on *kernelized score functions*, since it has been recently shown it is among the most competitive methods in the related problem of cancer module gene ranking [40], and on *random walks* algorithms, since they have been successfully applied to prioritize genes with respect to genetic diseases [19]. As a baseline method we used a simple implementation of the *guilt-by-association (GBA)* principle [56].

#### 2.5.1 Kernelized score functions

Kernel-based ranking methods have been recently proposed in the context of cancer module gene ranking [40], drug ranking [57] and gene function prediction problems [58,31]. Methods based on *kernelized score functions* are very fast (their time complexity is approximately linear in sparse graphs, once the kernel matrix is computed) [31], and their accuracy is at least comparable with state-of-the-art gene prioritization methods [40].

The score functions $S:V⟶{\mathbb{R}}^{+}$ are based on properly chosen kernels, by which we can directly rank vertices according to the values of *S*(*i*): the higher the score, the higher the likelihood that a gene belongs to a given MeSH disease.

*Kernelized score functions* rely on distance measures defined in a suitable Hilbert space H. More precisely, let *X* be a general nonempty set, ϕ:X→H, a mapping to a given universal reproducing kernel Hilbert space H, and K:X×X→ℝ its associated kernel function, such that $<\phi (\cdot ),\phi (\cdot ){>}_{H}=K(\cdot ,\cdot )$, where $<\cdot ,\cdot {>}_{H}$ represents the internal product in H. By choosing a distance measure on a Hilbert space, we can exploit the classical “kernel-trick” [59] and we can embed any valid kernel into the distance measure itself.

It is worth noting that we extend the notion of neighbour through the kernel *K*: by choosing an appropriate kernel, node *j* can be in the neighbour of node *i* even if there is no edge between them in the original graph *G*: i.e. $w_{\mathrm{ij}}=0$, but *K*(***x***_*i*_, ***x***_*j*_) > 0. From this standpoint the Gram matrix ***K*** can be interpreted as a novel “weighted adjacency matrix” in the projected Hilbert space induced by the mapping ϕ:X→H.

If we choose the minimum distance *D*_*NN*_ between *i* and *V*_*M*_ (the set of genes annotated for a given MeSH disease *M*), we can obtain the *nearest-neighbours score S*_*NN*_:

(12) $$D_{\mathrm{NN}}(i,V_{M})=\min_{j\in V_{M}}\frac{1}{2}∥\phi (x_{i})-\phi (x_{j}){∥}^{2}$$

By developing the square (12) we obtain:

(13) $$D_{\mathrm{NN}}(i,V_{M})=\min_{j\in V_{M}}\left[\frac{1}{2}<\phi (x_{i}),\phi (x_{i})>+\frac{1}{2}<\phi (x_{j}),\phi (x_{j})>-<\phi (x_{i}),\phi (x_{j})>\right]$$

By substituting in (13) the internal product <*ϕ*(·), *ϕ*(·) > with a suitable kernel *K*(· , ·), we can obtain a similarity measure simply by changing the sign:

(14) $${\mathrm{Sim}}_{\mathrm{NN}}(i,V_{M})=-\min_{j\in V_{M}}\left[\frac{1}{2}K(x_{i},x_{i})-K(x_{i},x_{j})+\frac{1}{2}K(x_{j},x_{j})\right]$$

If *K*(***x***_*j*_, ***x***_*j*_) are equal for all *j* ∈ *V*, we can simplify (14), thus achieving the *nearest neighbours score S*_*NN*_:

(15) $$S_{\mathrm{NN}}(i,V_{M})=-\min_{j\in V_{M}}-K(x_{i},x_{j})=\max_{j\in V_{M}}K(x_{i},x_{j})$$

A natural extension of the *S*_*NN*_ score can be obtained by introducing the *k-nearest neighbours distance*:

(16) $$D_{\mathrm{kNN}}(i,V_{M})=\frac{1}{2}\sum_{j\in I_{k}(i)}∥\phi (x_{i})-\phi (x_{j}){∥}^{2},$$

where *I*_*k*_(*i*) = {*j* ∈ *V*_*M*_|*j* isrankedamongthefirstkin *V*_*M*_}. By adopting a similar procedure used to derive the *S*_*NN*_ score, we can obtain from (16) the *k-nearest neighbours score S*_*kNN*_:

(17) $$S_{\mathrm{kNN}}(i,V_{M})=\sum_{j\in I_{k}(i)}K(x_{i},x_{j})$$

Using a distance *D*_*AV*_(*i*, *V*_*M*_) of a vertex *i* ∈ *V* with respect to a set of nodes *V*_*M*_, simply as the average distance in the Hilbert space between *i* and the set of nodes included in *V*_*M*_:

(18) $$D_{\mathrm{AV}}(i,V_{M})=\frac{1}{2}∥\phi (x_{i})-\frac{1}{\left|V_{M}\right|}\sum_{j\in V_{M}}\phi (x_{j}){∥}^{2}$$

we can derive from (18) the *average score S*_*AV*_:

(19) $$S_{\mathrm{AV}}(i,V_{M})=-\frac{1}{2}K(x_{i},x_{i})+\frac{1}{\left|V_{M}\right|}\sum_{j\in V_{M}}K(x_{i},x_{j})$$

This score represents the average similarity of the gene *i* with respect to the genes belonging to the set *V*_*M*_. If all *K*(***x***_*i*_, ***x***_*i*_) are equal for each *i* ∈ *V* (i.e. the “self-similarity” of genes does not matter), we can further simplify (19) by removing its first term.

Even if any valid kernel *K* can be applied to compute the above proposed scores, in the context of network-based gene prioritization, we used *random walk kernels* [53], since they can capture the similarity between genes, taking into account the topology of the overall functional interaction network.

The Gram matrix ***K*** associated to the *one-step random walk kernel* can be derived from the symmetric adjacency matrix ***W*** of the functional interaction undirected graph *G*:

(20) $$K=(a-1)I+D^{-\frac{1}{2}}WD^{-\frac{1}{2}}$$

where ***I*** is the identity matrix, ***D*** is a diagonal matrix with elements $d_{\mathrm{ii}}=\sum_{j}w_{\mathrm{ij}}$, and *a* is a value larger than 1.

The *q-step random walk kernels* ***K***_*q*−*step*_ = ***K***^*q*^, can be easily obtained by matrix multiplication from the one-step random walk kernel matrix (20), where *q* represents the number of random walk steps in the underlying graph [53]. In this way, by setting *q* = 2 or *q* = 3 two vertices are considered similar if they are directly connected or if they are connected through a path including one or two vertices. Also longer paths could be considered, by setting *q* > 3: in this way we can deeply explore the graph to find similarities between genes mediated through long paths in the graph.

#### 2.5.2 Random walks and random walks with restart

Kernelized score functions presented in the previous section can be interpreted as a generalization of the random walk algorithms, which have been successfully applied to gene prioritization problems [19,60]. Random walk (*RW*) algorithms [61] rank genes by exploring and exploiting the topology of the gene network: random walks across the network are performed starting from a subset *V*_*M*_ ⊂ *V* of genes belonging to a specific MeSH descriptor *M* by using a transition probability matrix ***Q*** = ***D***^−1^***W***, where ***W*** is the adjacency matrix, and ***D*** is a diagonal matrix with diagonal elements $d_{\mathrm{ii}}=\sum_{j}w_{\mathrm{ij}}$.

Starting from the initial set of probabilities ***p***_*o*_ of the genes 1 … *n* of belonging to *M*, where $p_{o}^{i}=1/V_{M}$ if *i* ∈ *V*_*M*_, otherwise $p_{o}^{i}=0$, the *RW* update rule:

(21) $$p_{t+1}=Q^{T}p_{t}$$

is repeated till to convergence or for a fixed number of iterations.

We can observe that the random walker could progressively “forget” the a priori information available for the MeSH descriptor *M*, by iteratively walking across the overall network. To avoid this problem, we can stop the *RW* algorithm after a few iterations, as outlined above, or we can apply the random walk with restart (*RWR*) method: at each step the random walker can move to one of its neighbours or can restart from its initial condition with probability *θ*:

(22) $$p_{t+1}=(1-\theta )Q^{T}p_{t}+\theta p_{o}$$

With both *RW* and *RWR* methods at the steady state we can rank the vector ***p*** to prioritize genes according to their likelihood to belong to the MeSH disease under study.

#### 2.5.3 Guilt by association methods

As a baseline gene prioritization method we applied a simple implementation of the *guilt-by-association (GBA)* principle. According to this general biological principle, a biomolecular entity that interacts or shares some features with another biomolecular entity can also share some specific biological property (for instance, its membership to a given MeSH category). In computational biology this basic biological principle has been exploited to develop methods able to assign a given biological or molecular property on the basis of the labeling of neighborhoods in biomolecular networks [56,62]. In the context of gene prioritization problems, we can assess the likelihood that a given gene belongs to a given MeSH category *M* on the basis of the M-labeled genes directly connected to the gene under study.

We implemented a simple version of the *GBA* approach, in which the score for each gene is computed by choosing the maximum of the weights $w_{\mathrm{ij}}\in W$ of the edges connecting the gene *i* to positive labeled genes *j* ∈ *V*_*M*_ in the neighborhood *N*(*i*) of *i*:

(23) $$S(i,M)=\max_{j\in N(i)}\;w_{\mathrm{ij}}$$

where *N*(*i*) = {*j*|*j* ∈ *V*_*M*_ ∧ (*i*, *j*) ∈ *E*}.

## 3 Results and discussion

### 3.1 Experimental set-up

One of the main goals of this work consists in performing an extensive analysis of gene-disease associations, considering a large set of diseases.

Moreover, we experimentally investigated the impact of network integration on gene prioritization, by performing a quantitative comparison of the accuracy achieved by the methods described in Section 2.5 using each of the single gene networks considered in Section 2.2 with that obtained through the network integration methods introduced in Section 2.4.

More precisely, at first we assessed the “informativeness” of each single gene network by analyzing the performance of *GBA*, *RW*, *RWR* and *kernelized score function* methods. Then we performed a systematic analysis of both *unweighted* and *weighted* network integration methods, by combining at first the six binary gene interaction networks and then by exploiting also the real-valued semantic similarity-based gene networks through the integration of all the available nine different nets (Table 1).

Moreover we indicated some unannotated genes as reliable “disease gene” candidates for a selected set of MeSH diseases for which we obtained robust and accurate predictions.

### 3.2 Evaluation of the gene prioritization and network integration methods

The generalization performances of each gene prioritization and network integration method has been assessed through a classical cross-validation procedure [63], setting to five the number of the folds. More precisely, the nodes of the graph have been randomly partitioned in five folds, and in turn a fold is selected as the test fold, while the remaining are the training folds. The labels of the test fold are removed, and the labels of the training folds are used to infer the scores to be assigned to the nodes of the test fold (in our setting we deal with gene prioritization, i.e. a ranking problem). Finally, having the scores predicted for each of the five folds (that is for the entire set of the available genes) we can apply standard measures to evaluate the correctness of the obtained gene ranking with respect to each disease. In particular we applied the AUC to evaluate the ranking of the genes. Moreover, we applied also the precision at a given recall to take into account that for several MeSH diseases we have a relatively low number of known disease genes (positive examples).

After the assessment of the generalization performance of the gene prioritization and network integration methods, we reported for each of the considered 708 MeSH diseases the *p*-value obtained through a non parametric statistical test based on the “shuffling” of the gene labels (Section 3.6). Then we reported the 10 top-ranked unannotated genes for each MeSH disease, and we performed also an analysis of the unannotated genes as reliable “disease gene” candidates on the basis of the distribution of the scores of the annotated genes for the MeSH diseases for which we obtained a very high estimated cross-validated AUC value.

We outline that the reported results are based, according to the literature on gene prioritization, on retrospective benchmarks, and for this reason offer usually optimistic estimates of the generalization performances, since disease-associations are likely to be directly or indirectly incorporated in the gene-prioritization data sources [3]. As outlined in [64], this problem is difficult to address in an initial study and can be resolved only by long-term perspective benchmarks, wherein predictions are made on the current state of knowledge (that is the current available annotations) and validated in future studies, that is once novel experimental evidence of disease-associations will be available.

### 3.3 Gene prioritization with single networks

We performed an assessment of the “informativeness” of each gene network through an extensive experimental evaluation of the average AUC results across 708 MeSH diseases, using different gene prioritization methods (Table 2). The first column of Table 2 shows the gene prioritization methods and their main associated learning parameters (see Section 2.5 for details). For each column the best average AUC results achieved by the gene prioritization methods are highlighted in bold. *S*_*AV*_ and *S*_*kNN*_ kernelized score functions achieve usually the best results, but also *RW* and *RWR* algorithms are sometimes comparable with kernelized score functions. The difference is statistically significant (Wilcoxon rank sum test, *α* = 0.01) in favor of kernelized score functions for the data sets *dbnet*, *finet*, *hnnet*, *bpnet* and *ccnet*, while for the other four functional networks no statistically significant difference has been detected.

The last row of Table 2 shows the average results across methods for each gene network. We can observe that on the average gene prioritization methods achieve the best results with *finet* and *gcnet*, but the AUC performances are relatively high also with *hnnet* and *bpnet*. The other nets appear to be less informative on the average, but consider that a certain learning is assured with each of the considered networks, since the average AUC is always significantly larger than 0.5.

It is not surprising that *finet*, *gcnet* (and also *hnnet*) are the most “informative” networks, since they are constructed by integrating different sources of information (Section 2.2). We only observe that with *gcnet* the results are referred only to a subset of the genes used in our experiments (Table 1). It is worth also noting the good results obtained with semantic similarity-based networks constructed from biological processes GO annotations (*bpnet*), even if also in this case the results are computed with respect to a subset of the *S* genes, and hence the comparison must be considered with a certain caution. Summarizing, the results witness for the fact that all the considered gene networks bear a certain information about the gene prioritization with MeSH diseases. In particular networks just constructed through the integration of different sources of evidence seem to be the most “informative” for this gene ranking task.

### 3.4 Gene prioritization with unweighted network integration

Our network integration experiments started with the combination of the six binary gene networks described in Section 2.2 (that is all the available gene networks excluding real-valued semantic similarity-based nets), using the unweighted combination methods presented in Section 2.4.2. Table 3 reports the average AUC results across MeSH diseases with *UA*, *PUA* and *MAX* integration methods. Note that we did not perform “soft” *MIN* integration since it is easy to see that with binary networks this method is indistinguishable from *MAX*, while “drastic” *MIN* leads to highly disconnected networks.

Comparing Tables 2 and 3, we can observe that unweighted integration improves the performance. This is true especially with *UA* and *PUA* methods (the difference is almost always statistically significant at *α* = 0.01 significance level), but in several cases also with *MAX*. The improvement depends also on the gene prioritization method used. For instance unweighted integration degrades performance with *S*_*NN*_ (at least with respect to the most informative single gene networks), while with the other kernelized score functions and with *GBA*, *RW* and *RWR* algorithms often unweighted integration improves AUC results. While a larger number of steps improves the performance of kernelized score functions, with the classical *RW* algorithm we observe a degradation of the performances. These results show that the classical *RW* tends to “forget” the initial “a priori” knowledge, while kernelized score functions retain the prior information and are able to exploit the overall topology of the network, confirming previous results [40,31].

Hereinafter we limited the integration experiments to kernelized score functions only, since they usually perform equally or better than the other compared methods, and their empirical time complexity is significantly lower than *RW* and *RWR* algorithms: for instance, while an entire cycle of cross-validation on the 708 MeSH classes with *UA* integration requires hours with *RWR*, the same task requires only some minutes with kernelized score functions, using an Intel i7 2.80 GHz processor with 16 GB of RAM and a Linux system.

By adding the real-valued networks based on semantic similarity measures (Section 2.2), we observe a further significant enhancement of the overall performance, showing that the integration of different sources of evidence leads to better results (Table 4). For instance the performances of the *UA* approach with *S*_*AV*_ using a five step random walk kernel are boosted from 0.8596 to 0.8831 average AUC (the increment is significant at *α* = 10^−30^ significance level according to the Wilcoxon signed rank sum test). Note that the *MIN* integration fails on this task, since an “intersection” strategy in this context leads to a significant loss of information, thus not allowing to exploit the topological information underlying the entire network.

**Fig. 3:**
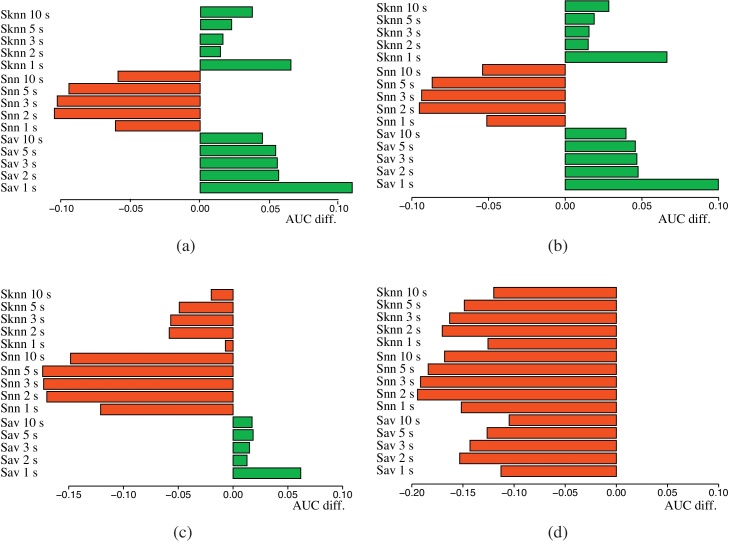
Unweighted integration methods: differences of average AUC across MeSH diseases with respect to the best single gene network (*finet*). (a) *UA* (b) *PUA* (c) *MAX* (d) *MIN*.

Fig. 3 provides a visual clue of the differences of average AUC across MeSH categories between unweighted integration methods and the best single gene network (*finet*). Fig. 3(d) confirms that also in this task *MIN* integration fails, for the same reasons explained above. On the contrary *UA* and *PUA* integration provides significant enhancements with both *S*_*AV*_ and *S*_*kNN*_ (Fig. 3(a) and (b)). Note that unweighted integration with *S*_*NN*_ results in a degradation of the performances (Fig. 3). We have not a clear explanation of this fact, but we think that the instability of scores computed by using only one of the neighbours, combined with the impossibility of weighting or choosing the best sources of information, may add noise to the prediction process.

Summarizing, the results show that unweighted integration, and especially *UA* and *PUA* methods, significantly enhances gene prioritization results. All the considered gene prioritization methods, ranging from random walks to kernelized score functions (with the exception of *S*_*NN*_), derive a benefit from unweighted integration. Moreover, the integration of semantic similarity-based networks further improves the performances of gene prioritization. Note that with these networks, considered individually, gene prioritization methods do not attain high average AUC scores (at least with *mfnet* and *ccnet*, Table 2), but their integration significantly enhance gene prioritization results (Table 4), since they convey complementary information with respect to the other sources of evidence.

### 3.5 Gene prioritization with weighted network integration

We experimented also with *WA* and *WAP* network integration to explicitly take into account the “informativeness” of each gene network (Section 2.4.3). Table 5 shows that weighted integration significantly boosts the performance of kernelized score functions. In particular five-steps *S*_*AV*_ with weighted integration of all the nine available nets (*WA-all*, Table 5) reaches the highest AUC average score, but almost all the gene prioritization algorithms achieve their best results with *WA* and *WAP* integration.

**Fig. 4:**
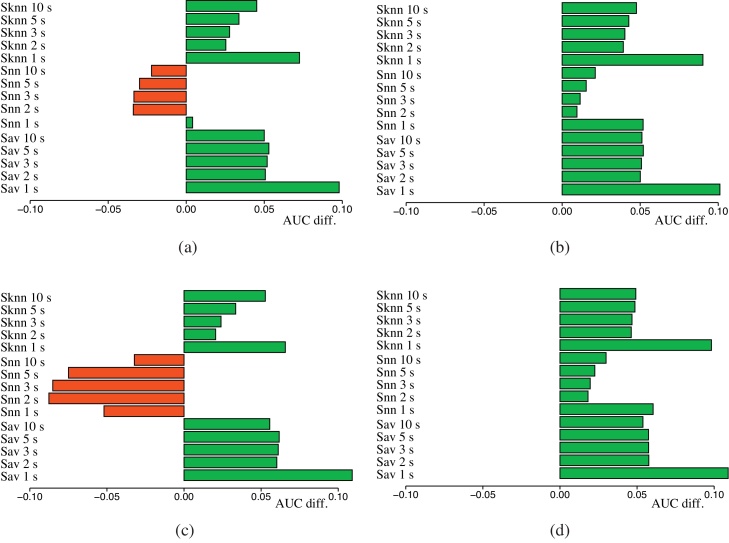
Weighted integration methods: differences of average AUC across MeSH categories with respect to the best single gene network (*finet*). Integration of six networks: (a) *WA* (b) *WAP*. Integration with nine networks including semantic similarity-based nets: (c) *WA* (d) *WAP*.

This is more evident in Fig. 4, where we register a very high increment of the average AUC score with respect to the best single gene network. This is true for both *S*_*AV*_ and *S*_*kNN*_, while for *S*_*NN*_ this behavior is limited to *WAP* methods only (Fig. 4(b) and (d)). Nevertheless, note that, on the contrary, *S*_*NN*_ behaves badly with unweighted integration, independently of the combination method applied (Table 3).

**Fig. 5:**
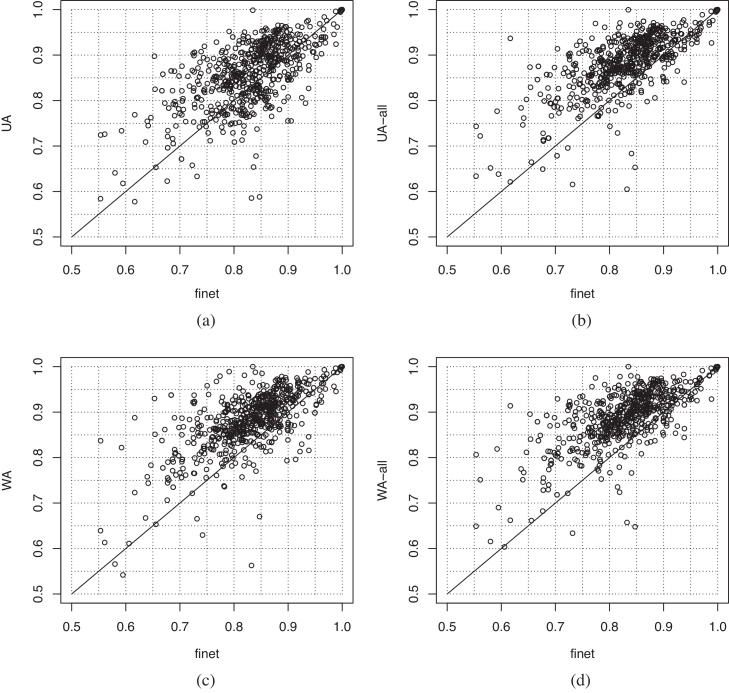
Comparison of AUC results between network integration methods and the best single gene network (*finet*). Each point represents the AUC score obtained by *S*_*AV*_ five steps with network integration methods (ordinate) and with the best single network *finet* (abscissa) on each of the 708 MeSH diseases. (a) *UA* with six networks; (b) *UA* with all the nine networks; (c) *WA* with six networks; (d) *WA* with all nine networks.

To get more insights into the results obtained with unweighted and weighted integration methods, Fig. 5 compares the AUC scores for each class achieved by five steps *S*_*AV*_ (one of the best gene prioritization method) between unweighted and weighted integration with respect to the the best single network *finet*. A point in Fig. 5 represents the AUC score, relative to a MeSH disease, attained by the integration method and by the best single gene network. More precisely, the AUC value obtained by the integration method is represented in ordinate, while in abscissa we have the AUC value achieved with *finet*, i.e. the best single network. Points that lie above the bisector of the first quadrant angle represent MeSH diseases for which the integration method achieves better results than the single best gene network. In Fig. 5(a) most of the points lie above the bisector, showing that *UA* enhances results obtained with *finet*. By adding semantic similarity-based gene networks several points moves above the bisector line (Fig. 5(b)), confirming that these networks add novel useful information for the gene prioritization task. Looking at Fig. 5(c) we observe that with *WA* integration, just without semantic similarity-based gene networks, most of the points lie above the bisector, and the results are also better when we integrate all the available networks (Fig. 5(d)).

**Fig. 6:**
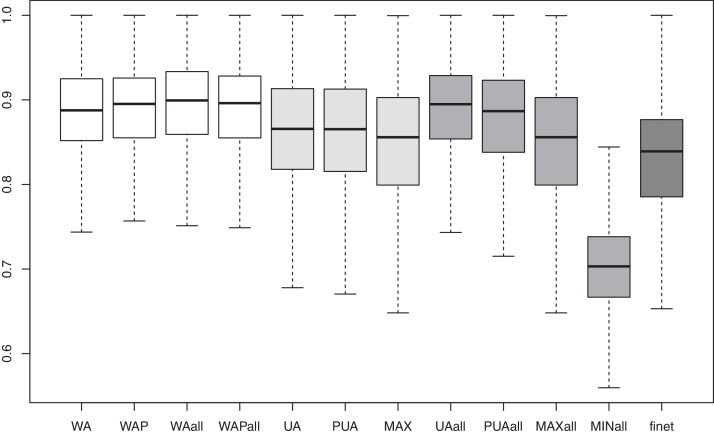
Boxplots of the AUC results of the different network integration methods. The last boxplot on the rights refers to the best result with a single network (*finet*).

Fig. 6 provides an overall picture of the distributions of AUC scores compared between different unweighted and weighted integration methods using five steps *S*_*AV*_ as gene prioritization algorithm. White boxplots refer to weighted integration methods, light gray boxplots to unweighted integration methods without semantic similarity-based gene networks, and dark gray boxplots to unweighted methods integrating all the nine available gene networks. Weighted methods show the best results (especially when all the networks are integrated), but also *UAll*, that is *UA* integrating all the available nine nets, achieve quite similar results. All the considered methods behave better than the best single gene network (last boxplot in Fig. 6), except for *MIN*, that clearly fails on this task, as just discussed above.

To obtain a more reliable comparison of the results obtained with different gene network integration methods, we applied to each pair of them the Wilcoxon signed rank sum test, to estimate whether a significant statistical difference does exist using the best performing gene prioritization method (*S*_*AV*_ five steps). Table 6 summarizes the main results: a “+” entry means that a significant statistical difference at 0.01 significance level is registered in favor of the method in the row with respect to the method in the column; a “−” entry means that the opposite holds, and a “=” entry stands for no significant difference between the methods.

We observe that weighted integration is always significantly better or equal than all the other compared methods. In particular *WA-all* integration (that is, *WA* integrating all the available nets) is significantly better than all the other considered integration approaches. Note that also *UA-all* is always better or equal than all the others (except with *WA-all*), showing that also a simple unweighted integration, if a sufficiently large set of sources of evidence is provided, can achieve results comparable with the more computationally expensive weighted integration (recall that the weights of the integration are obtained by evaluating the AUC on each single gene network by internal cross-validation, see Section 2.4.3). Quite interestingly, *WAP* does not outperform *WA*: even if we construct a specific weighted network for each MeSH disease this does not introduce a significant advantage (at least, on the average). This fact could be explained by considering that the per-class integration (*WAP*) may introduce a certain overfitting to the data, while *WA*, by averaging the weights across classes and thus resulting in a single integrated network, could reduce the overfitting, acting as a sort of “regularization”, confirming previous results obtained in the context of gene function prediction [55].

**Fig. 7:**
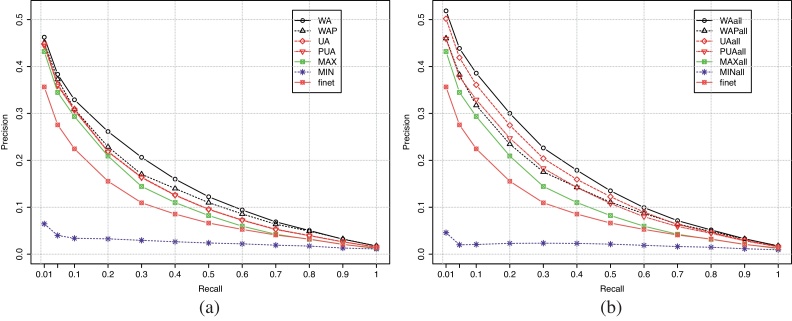
Comparison of the average precision at fixed levels of recall across the 708 MeSH diseases between network integration methods and the best single gene network (*finet*). (a) Results relative to the integration of the basic six networks; (b) results with all the integrated nine networks, including semantic similarity-based nets.

Considering that for a large number of diseases we have a relatively low number of annotated genes, we compared also the precision at different recall levels between different unweighted and weighted integration methods, using two steps *S*_*AV*_ as gene prioritization algorithm (Fig. 7). With both the integration of the six basic networks (Fig. 7 (a)) and with the integration of the six basic networks plus the three semantic similarity-based networks (Fig. 7(b)) we achieve significantly better results with any of the considered integrated network with respect to the best “single” network (*finet*), except for the *MIN* integration that obtains the worst results. Also in this case weighted integration outperforms unweighted integration, but observe that when we integrate all the available networks *UAall*, i.e. the unweighted average integration, achieves better results than the weighted per-class integration (*WAPall*), confirming that *WAP* integration undergoes a certain overfitting to the data. Note that when semantic-similarity based networks are added, all the integration methods improves their precision/recall results (the scale of the ordinate, that is the precision is equal in Fig. 7(a) and (b)). For instance *WA*, the best performing network integration methods, improves its average precision at 20% recall from 0.26 to 0.30 with a relative increment of about 15% in precision. As a final observation, note that all the considered network integration methods (except *MIN* integration) significantly outperform the results obtained with the best single network, confirming that also simple unweighted integration algorithms are sufficient to boost the performance of gene prioritization methods.

### 3.6 Finding novel associations between genes and MeSH diseases

The common usage of genes ranking scores in gene-disease prioritization experiments consists in the selection of the top ranked unannotated genes and in the their further characterization as possible “candidate” genes actually implied in the onset and progression of the considered disease.

To this end we provide for each of the 708 MeSH diseases the AUC obtained by five-fold cross-validation, the *p*-value achieved through a non parametric randomized test (see below), and the 10 top ranked genes currently not annotated for the MeSH disease under study. Table summarizing these information is available at http://homes.di.unimi.it/re/suppmat/genesmeshnetwpred/supmatTBL1.html (accessed 30 November 2013).

Moreover, we also provide a preliminary analysis of the top ranked most reliable unannotated genes for the MeSH diseases predicted with high robustness and accuracy by the best network integration, i.e. *WA* integrating all the available nets using five steps *S*_*AV*_ to prioritize genes.

**Fig. 8:**
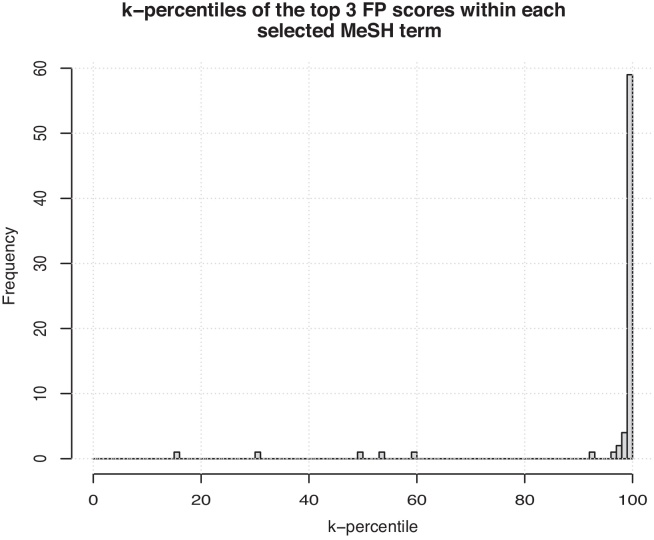
Frequency of the *k*-percentiles of the three top ranked unannotated genes.

To evaluate the robustness of the method we performed a non-parametric statistical test by randomly shuffling 1000 times the labels for each MeSH disease and counting how many times *m* the AUC computed with randomly shuffled labels is larger than the AUC computed with the true labels. The resulting p-value is just the ratio $\frac{m}{1000}$. Interestingly enough, we achieve a *p*-value < 0.01 for 649 and a p-value <0.05 for 676 of the 708 MeSH diseases. To choose MeSH diseases both robustly and accurately predicted we selected MeSH descriptors with an average AUC ≥0.975 and *p*-value<0.01, resulting in a set of 24 diseases. For each of the selected diseases, we extracted the lowest score *c* from the set of positive (annotated) genes. Then, we computed the empirical cumulative distribution of all the scores equal or larger than *c*, considering both annotated and unannotated genes. As a final step, using the distribution computed at the previous step, we computed the *k*-percentiles of the three top ranked unannotated genes within each selected MeSH term. Considering that we selected 24 MeSH diseases, this procedure lead to a collection of 72 *k*-percentiles whose frequency is plotted in Fig. 8.

Fig. 8 shows that most of the top ranked unannotated genes are concentrated close to the 100-percentile, showing that these top ranked “false positive” genes are “strongly predicted” as possible candidate disease genes, since their scores are close to that of the top ranked annotated genes. Consider also that this is supported by the fact that we selected only diseases for which gene prioritization achieved a very high AUC and “robust” predictions (*AUC* > 0.975 and *p*-value <0.01). The top three false positives gene symbols along with the disease identifiers and disease names for the selected 24 MeSH descriptors are listed in Table 7.

Of course the proposed top ranked genes are only disease gene candidates, and these results need to be biologically interpreted and should undergo a rigorous bio-medical analysis prior to be actually associated to the disease itself.

## 4 Conclusions

We performed an extensive analysis of gene-disease associations not limited to genetic disorders, including more than 700 MeSH diseases.

By using network integration and gene prioritization methods, we reported for each disease the 10 unannotated top-ranked genes, available for further bio-medical analysis. Moreover, by analyzing the top-ranked predictions relative to the 24 best and robustly predicted MeSH diseases, we showed that our approach can detect reliable candidate disease genes.

It is well-known that the integration of multiple omics sources of evidence is of paramount importance in several application domains in computational biology [65–68]. In this work we performed a systematic comparison of unweighted integration and our proposed weighted combination methods to provide an evaluation of the impact of network integration on gene prioritization. We quantitatively showed that network integration is necessary to boost gene prioritization results, according to previous results published in the literature [15,69,27,28,46,47].

In particular, we showed that the proposed weighted integration methods, by exploiting the different “informativeness” embedded in different gene interaction networks, significantly outperform unweighted integration. Moreover our experimental results show that the performances strongly depend on the selection of the sources of evidence and on the characteristics of the gene networks. For instance, also a simple *UA* integration can significantly improve the performance of gene prioritization methods if a sufficient number of diverse and complementary gene interaction networks are combined. From this standpoint, a novel research line could be represented by an adaptation of test and select methods, originally proposed in the context of supervised ensembles [70] to appropriately choose the most predictive sources of evidence and gene networks for each MeSH disease through an adaptive learning process.

Confirming previous results [30], semantic similarity-based networks, combined with other sources of evidence boost the performance of gene prioritization methods. A possible improvement of the proposed approach could consist in combining networks based on semantic similarity measures that embed the ontology beneath the GO terms and are able to model the annotation uncertainty, according to the approach proposed in [45].

Quite surprisingly *WAP* does not outperform *WA* integration: this is likely due to overfitting, confirming previous results obtained in the context of gene function prediction [55].

Finally, our results show that *S*_*AV*_ kernelized score functions with five-steps random walk kernels using *WA* integration significantly outperform all the other considered methods. This means that in order to boost gene prioritization we need: (a) gene prioritization algorithms able to exploit the overall topology of the network; (b) weighted integration methods, able to learn from the data how to combine different gene interaction networks.

These results suggest novel research lines able to combine network integration methods, that learn from the data how to weight multiple sources of evidence, with network-based ranking algorithms that can learn from the overall topology of the integrated network how to prioritize candidate disease genes.

Recalling that we analyzed relatively simple network integration methods, a possible development of this work could consist in the comparative analysis of other more complex network integration approaches.

## Figures and Tables

**Table 1:** Characteristics of the gene networks used in our experiments.

Network	Description	Type	Nodes	Edges	Density
*finet*	Obtained from multiple sources of evidence	Binary	8449	271466	0.0038
*hnnet*	Obtained from multiple sources of evidence	Binary	8449	502222	0.0070
*cmnet*	Network projections from cancer modules	Binary	8449	3414722	0.0478
*gcnet*	Network projections from CTD	Binary	7649	1421298	0.0242
*bgnet*	Network projections from BioGRID	Binary	8449	120169	0.0016
*dbnet*	Direct relationships obtained from BioGRID	Binary	8449	3023084	0.0423
*bpnet*	Semantic similarity network from GO BP	Real valued	6923	44506147	0.9286
*mfnet*	Semantic similarity network from GO MF	Real valued	6145	26611887	0.7047
*ccnet*	Semantic similarity network from GO CC	Real valued	6693	39652637	0.8851

**Table 2:** Single gene networks: AUC results averaged across 708 MeSH diseases. The last row shows the average results across methods for each gene network.

	*cmnet*	*bgnet*	*dbnet*	*finet*	*hnnet*	*gcnet*	*bpnet*	*mfnet*	*ccnet*
*GBA*	0.6620	0.6389	0.6683	0.7542	0.7323	0.7346	0.7134	0.6395	0.6250
*RW* 1 step	**0.6922**	**0.6590**	0.6037	0.7356	0.7269	0.8418	0.7646	0.6985	0.6845
*RW* 2 step	0.6829	0.6462	0.6761	0.8194	0.7802	0.8220	0.7635	0.7013	0.6812
*RW* 3 step	0.6768	0.6406	0.6531	0.8157	0.7531	0.8145	0.7611	0.6985	0.6745
*RW* 5 step	0.6718	0.6316	0.6426	0.7993	0.6973	0.8089	0.7610	0.6834	0.6711
*RW* 10 step	0.6694	0.6224	0.6222	0.7575	0.6249	0.8075	0.7411	0.6790	0.6684
*RWR* *θ* = 0.6	0.6871	0.6515	0.6781	0.8271	0.7889	0.8401	0.7825	0.7112	0.6856
*RWR* *θ* = 0.9	0.6878	0.6513	0.6750	0.8242	0.7870	0.8453	0.7789	0.7085	0.6825
*S*_*AV*_ 1 step	0.6894	0.6574	0.6717	0.7669	0.7596	0.8167	**0.7889**	0.7139	**0.6916**
*S*_*AV*_ 2 step	0.6842	0.6414	0.6831	0.8226	0.7872	0.8328	0.7888	0.7142	0.6914
*S*_*AV*_ 3 step	0.6845	0.6417	0.6752	0.8255	0.7897	0.8417	0.7879	0.7146	0.6913
*S*_*AV*_ 5 step	0.6850	0.6418	0.6778	0.8287	0.7943	**0.8471**	0.7839	**0.7151**	0.6907
*S*_*AV*_ 10 step	0.6849	0.6408	0.6804	**0.8312**	0.7983	0.8407	0.7640	0.7117	0.6882
*S*_*NN*_ 1 step	0.6296	0.6263	0.6667	0.7561	0.7374	0.7308	0.6971	0.6485	0.6565
*S*_*NN*_ 2 step	0.6235	0.6105	0.6764	0.8031	0.7624	0.7316	0.7032	0.6478	0.6567
*S*_*NN*_ 3 step	0.6228	0.6105	0.6683	0.8044	0.7638	0.7365	0.7103	0.6475	0.6574
*S*_*NN*_ 5 step	0.6213	0.6107	0.6708	0.8052	0.7674	0.7481	0.7280	0.6475	0.6593
*S*_*NN*_ 10 step	0.6197	0.6136	0.6744	0.8029	0.7729	0.7774	0.7703	0.6493	0.6659
*S*_*kNN*_ 1 step *k* = 3	0.6439	0.6336	0.6705	0.7635	0.7523	0.7370	0.7645	0.6812	0.6712
*S*_*kNN*_ 2 step *k* = 3	0.6377	0.6179	0.6817	0.8149	0.7788	0.7403	0.7705	0.6937	0.6725
*S*_*kNN*_ 3 step *k* = 3	0.6371	0.6183	0.6737	0.8168	0.7805	0.7482	0.7765	0.6999	0.6756
*S*_*kNN*_ 5 step *k* = 3	0.6362	0.6191	0.6763	0.8182	0.7845	0.7647	0.7815	0.7003	0.6788
*S*_*kNN*_ 10 step *k* = 3	0.6366	0.6225	0.6798	0.8172	0.7898	0.7993	0.7695	0.7021	0.6803
*S*_*kNN*_ 1 step *k* = 19	0.6811	0.6523	0.6717	0.7668	0.7596	0.7860	0.7702	0.6997	0.6798
*S*_*kNN*_ 2 step *k* = 19	0.6756	0.6364	**0.6831**	0.8222	0.7871	0.8004	0.7763	0.7001	0.6799
*S*_*kNN*_ 3 step *k* = 19	0.6755	0.6368	0.6752	0.8249	0.7895	0.8125	0.7819	0.7008	0.6801
*S*_*kNN*_ 5 step *k* = 19	0.6757	0.6373	0.6779	0.8276	0.7940	0.8286	0.7902	0.7025	0.6807
*S*_*kNN*_ 10 step *k* = 19	0.6766	0.6373	0.6810	0.8292	**0.7986**	0.8402	0.7774	0.7063	0.6820

**Average**	0.6625	0.6338	0.6691	0.8029	0.7657	0.7955	0.7624	0.6899	0.6751

**Table 3:** Unweighted integration of the six binary gene networks (without semantic similarity-based nets): AUC results averaged across 708 MeSH categories.

	*UA*	*PUA*	*MAX*
*GBA*	0.8313	0.8291	0.6589
*RW* 1 step	0.8566	0.8563	0.8501
*RW* 2 step	0.8186	0.8178	0.8154
*RW* 3 step	0.7937	0.7925	0.7897
*RW* 5 step	0.7773	0.7760	0.7746
*RW* 10 step	0.7720	0.7704	0.7706
*RWR* *θ* = 0.6	0.8533	0.8528	**0.8520**
*RWR* *θ* = 0.9	0.8565	0.8531	0.8476
*S*_*AV*_ 1 step	0.8538	0.8530	0.8286
*S*_*AV*_ 2 step	0.8562	0.8554	0.8353
*S*_*AV*_ 3 step	0.8580	0.8571	0.8405
*S*_*AV*_ 5 step	**0.8596**	**0.8587**	0.8470
*S*_*AV*_ 10 step	0.8548	0.8540	0.8485
*S*_*NN*_ 1 step	0.6934	0.6921	0.6352
*S*_*NN*_ 2 step	0.6950	0.6936	0.6331
*S*_*NN*_ 3 step	0.6968	0.6954	0.6315
*S*_*NN*_ 5 step	0.7020	0.7004	0.6314
*S*_*NN*_ 10 step	0.7251	0.7230	0.6546
*S*_*kNN*_ 1 step *k* = 3	0.7280	0.7266	0.6593
*S*_*kNN*_ 2 step *k* = 3	0.7304	0.7289	0.6581
*S*_*kNN*_ 3 step *k* = 3	0.7332	0.7317	0.6580
*S*_*kNN*_ 5 step *k* = 3	0.7405	0.7389	0.6627
*S*_*kNN*_ 10 step *k* = 3	0.7636	0.7616	0.6987
*S*_*kNN*_ 1 step *k* = 19	0.8138	0.8124	0.7598
*S*_*kNN*_ 2 step *k* = 19	0.8170	0.8155	0.7639
*S*_*kNN*_ 3 step *k* = 19	0.8199	0.8183	0.7680
*S*_*kNN*_ 5 step *k* = 19	0.8251	0.8233	0.7785
*S*_*kNN*_ 10 step *k* = 19	0.8374	0.8356	0.8093

**Table 4:** Unweighted integration methods: AUC results averaged across 708 MeSH categories including all the available nine gene networks

	*UA-all*	*PUA-all*	*MAX-all*	*MIN-all*
*S*_*AV*_ 1 step	0.8765	0.8667	0.8286	0.6541
*S*_*AV*_ 2 step	0.8792	0.8701	0.8353	0.6694
*S*_*AV*_ 3 step	0.8811	0.8722	0.8405	0.6824
*S*_*AV*_ 5 step	**0.8831**	**0.8744**	0.8470	0.7023
*S*_*AV*_ 10 step	0.8761	0.8708	**0.8485**	**0.7264**
*S*_*NN*_ 1 step	0.6950	0.7050	0.6352	0.6045
*S*_*NN*_ 2 step	0.6980	0.7080	0.6331	0.6087
*S*_*NN*_ 3 step	0.7014	0.7108	0.6315	0.6129
*S*_*NN*_ 5 step	0.7106	0.7185	0.6314	0.6212
*S*_*NN*_ 10 step	0.7437	0.7490	0.6546	0.6349
*S*_*kNN*_ 1 step *k* = 19	0.8322	0.8331	0.7598	0.6413
*S*_*kNN*_ 2 step *k* = 19	0.8368	0.8372	0.7639	0.6520
*S*_*kNN*_ 3 step *k* = 19	0.8413	0.8404	0.7680	0.6619
*S*_*kNN*_ 5 step *k* = 19	0.8500	0.8465	0.7785	0.6789
*S*_*kNN*_ 10 step *k* = 19	0.8665	0.8576	0.8093	0.7093

**Table 5:** Weighted integration methods: AUC results averaged across 708 MeSH categories. *WA* and *WAP* include only the first six functional networks, while *WA-all* and *WAP-all* include all the nine functional networks.

	*WA*	*WAP*	*WA-all*	*WAP-all*
*S*_*AV*_ 1 step	0.8649	0.8680	0.8778	0.8768
*S*_*AV*_ 2 step	0.8733	0.8727	0.8828	0.8802
*S*_*AV*_ 3 step	0.8774	0.8763	0.8866	0.8830
*S*_*AV*_ 5 step	**0.8817**	0.8807	**0.8904**	**0.8861**
*S*_*AV*_ 10 step	0.8812	**0.8823**	0.8868	0.8850
*S*_*NN*_ 1 step	0.7602	0.8080	0.7042	0.8165
*S*_*NN*_ 2 step	0.7692	0.8126	0.7155	0.8213
*S*_*NN*_ 3 step	0.7709	0.8159	0.7193	0.8240
*S*_*NN*_ 5 step	0.7753	0.8206	0.7303	0.8278
*S*_*NN*_ 10 step	0.7807	0.8241	0.7707	0.8328
*S*_*kNN*_ 1 step *k* = 19	0.8394	0.8570	0.8325	0.8650
*S*_*kNN*_ 2 step *k* = 19	0.8476	0.8614	0.8427	0.8684
*S*_*kNN*_ 3 step *k* = 19	0.8527	0.8651	0.8489	0.8716
*S*_*kNN*_ 5 step *k* = 19	0.8614	0.8703	0.8611	0.8762
*S*_*kNN*_ 10 step *k* = 19	0.8744	0.8768	0.8819	0.8784

**Table 6:** Comparison between network integration methods: methods whose AUC performance are significantly better are marked with “+”, significantly worse with “−−” and with no significant difference with “=” (0.01 significance level, Wilcoxon signed rank sum test). The comparisons are in the sense rows vs. columns.

	*WAP-all*	*WA*	*WAP*	*UA-all*	*PUA-all*	*MAX-all*	*MIN-all*	*UA*	*PUA*	*MAX*	*finet*
*WA-all*	+	+	+	+	+	+	+	+	+	+	+
*WAP-all*		=	=	=	+	+	+	+	+	+	+
*WA*			=	=	+	+	+	+	+	+	+
*WAP*				=	+	+	+	+	+	+	+
*UA-all*					+	+	+	+	+	+	+
*PUA-all*						+	+	+	+	+	+
*MAX-all*							+	−	−	=	+
*MIN-all*								−	−	−	−
*UA*									+	+	+
*PUA*										+	+
*MAX*											+

**Table 7:** List of 24 selected diseases and of the corresponding top ranked unannotated genes.

Disease id.	Disease name	Top ranked unannotated genes
C535579	Cardiofaciocutaneous syndrome	KSR2,PILRA,KSR1
C536436	Coffin-Siris syndrome	PYGO1,ARID2,SMARCC2
C536664	Peroxisome biogenesis disorders	PEX5,PEX7,LONP2
C536783	T-Lymphocytopenia	BIRC8,CASP10,NAIP
C536928	Turcot syndrome	MLH3,PMS2L5,MSH3
C537345	Sitosterolemia	UGT1A5,UGT2B17,SLCO1B1
C538169	Acitretin embryopathy	CASP10,PEA15,SLCO3A1
D000562	Amebiasis	DCLRE1C,IL19,CYP2C8
D001404	Babesiosis	DCLRE1C,IL19,FCGR2C
D002062	Bursitis	UGT2B4,UGT2B15,UGT1A4
D006958	Hyperostosis, Cortical, Congenital	NPPC,NPR1,ACE
D007888	Leigh Disease	NDUFB10,NDUFB4,NDUFA12
D008118	Loiasis	FCGR2C,CYP3A43,CYP8B1
D008375	Maple Syrup Urine Disease	ACAD8,PDHX,PDHB
D009196	Myeloproliferative Disorders	PTPN1,CISH,SLC25A40
D009634	Noonan Syndrome	KSR2,KSR1,MRAS
D010483	Periapical Diseases	MMP13,IL12B,IL8
D012214	Rheumatic Heart Disease	CYP21A2,CYP8B1,CYP3A43
D014353	Trypanosomiasis, African	DCLRE1C,BCL2,STAT1
D015823	Acanthamoeba Keratitis	DCLRE1C,IL19,CYP2C8
D018235	Smooth Muscle Tumor	NFKB1,IL8,IL6
D020299	Intracranial Hemorrhage, Hypertensive	NPPC,NPPB,CRH
D056685	Costello Syndrome	KSR2,PILRA,KSR1
D056824	Upper Extremity Deep Vein Thrombosis	FGGCX,PROZ,F11
